# *In vitro* Evaluation of the Effect of Combination of Hydrophilic and Hydrophobic Polymers on Controlled Release Zidovudine Matrix Tablets

**DOI:** 10.4103/0250-474X.44594

**Published:** 2008

**Authors:** S. Ganesh, M. Radhakrishnan, M. Ravi, B. Prasannakumar, J. Kalyani

**Affiliations:** Department of Pharmaceutics, the Erode College of Pharmacy, Erode-638 112, India

**Keywords:** HPMC K4 M, Carbopol, matrix tablet, zidovudine, controlled release

## Abstract

The aim of the present study was to prepare and characterize controlled-release matrix tablets of zidovudine using hydrophilic HPMC K4 M or Carbopol 934 alone or in combination with hydrophobic ethyl cellulose. Release kinetics was evaluated by using USP XXIV dissolution apparatus No.2 (paddle) type. Scanning electron microscopy was used to visualize the effect of dissolution medium on matrix tablet surface. The *in vitro* results of controlled – release zidovudine tablets were compared with conventional marketed tablet Zidovir. The *in vitro* drug release study revealed that HPMC K4 M or Carbopol 934 preparation was able to sustain the drug release near to 6 hours. Combining HPMC K4 M or Carbopol 934 with ethyl cellulose sustained the drug release for nearly 12 h. The *in vitro* evaluation showed that the drug release may be by diffusion along with erosion. Results suggest that the developed controlled-release tablets of zidovudine could perform therapeutically better than marketed dosage forms, leading to improve efficacy, controlling the release and better patient compliance.

HIV (Human Immunodeficiency Virus) is a virus that causes AIDS (Acquired Immunodeficiency Syndrome), a health condition in which a person is affected by a series of diseases because of poor immunity. It is crucial for the success of AIDS therapy to maintain the drug concentration consistently above its target antiretroviral concentration throughout the course of the treatment^1^.

Zidovudine (AZT) (3’-azido-3’-deoxythymidine) is a thymidine analogue in which the 3- hydroxyl group is replaced by an azido(-N_3_) group. It is the first antiHIV compound approved for clinical use in the treatment of AIDS either alone or in combination with other antiviral agents. The main limitation to therapeutic effectiveness of AZT is its dose-dependent hematological toxicity, low therapeutic index, short biological half-life, and poor bioavailability. It is rapidly absorbed from the gastrointestinal tract (GIT) exhibiting a peak plasma concentration of 1.27 μM. The biological half-life of AZT-triphosphate is 3-4 h, thus necessitating frequent administration (3 to 4 times a day) to maintain constant therapeutic drug levels. Since its antiviral effect is time dependent, an adequate zero-order delivery of AZT is desired for maintaining antiAIDS effect and avoiding the toxic side effects like granulocytopenia and severe anemias usually associated with excessive plasma level of AZT immediately after intravenous or oral administration.

AZT is completely and rapidly absorbed throughout the GI Tract with a bioavailability of 65%. As the drug is freely soluble at all pH, judicious selection of release-retarding excipients is necessary for achieving constant *in vivo* release. Number of studies shows the use of hydrophilic matrices to formulate the controlled release dosage forms of different drugs 2–9. Because of their simplicity and cost-effectiveness, hydrophilic gel matrix tablets are widely used for oral controlled release dosage forms. Hydrophilic polymers form a gel like structure around the tablet core which controls the drug release. The hydrophilic polymers, HPMC K4 M or Carbopol 934^10^ selected in the present study provide pH-independent drug release to oral dosage forms that can be used for formulating the sustained-release dosage forms. However, the use of hydrophilic polymer alone for controlling the drug release of highly water soluble drugs is restricted due to rapid diffusion of the dissolved drug through the hydrophilic gel layer. Use of hydrophobic polymers will retard the drug release. So in the present investigation, an attempt has been made to formulate the controlled – release matrix tablets of AZT using hydrophilic matrix material (HPMC K4 M or Carbopol 934) in combination with hydrophobic ethyl cellulose^4^,^11^,^12^.

## MATERIALS AND METHODS

AZT was obtained as a gift sample from Aurobindo Pharma Ltd., (Hyderabad, India). HPMC K4 M, Carbopol 934 and ethyl cellulose were purchased from Merck Chemicals (Mumbai). All other chemicals and reagents used in the study were of analytical grade and were used as procured.

### Preparation of matrix tablets:

Matrix tablets were prepared by direct compression technique using micro crystalline cellulose as directly compressible vehicle and magnesium stearate as lubricant. All the ingredients were sieved through 40 mesh screen and mixed with other ingredients and the powder mixture was compressed using 7 mm round, flat and plain punches on multiple punch rotary tablet machine. In total, 10 formulations containing different amounts of HPMC K4 M (F1, F2, F3), Carbopol 934 (F4, F5, F6) and combination of HPMC K4 M or Carbopol 934 with ethyl cellulose (F7, F8, F9, F10) were prepared and their formulae are presented in Table 1.

**TABLE 1:** FORMULAE OF ZIDOVUDINE MATRIX TABLETS

Ingredients	F1 (mg)	F2 (mg)	F3 (mg)	F4 (mg)	F5 (mg)	F6 (mg)	F7 (mg)	F8 (mg)	F9 (mg)	F10 (mg)
Zidovudine	300	300	300	300	300	300	300	300	300	300
HPMC K4 M	91.5	122	153	-	-	-	76.5	-	61.8	-
Carbopol 934	-	-	-	91.5	122	153	-	76.5	-	61.8
Ethyl cellulose	-	-	-	-	-	-	76.5	76.5	91.2	91.2
Microcrystalline	211.5	181	150	211.5	181	150	150	150	150	150
Cellulose										
Magnesium	7	7	7	7	7	7	7	7	7	7
stearate										
Total (mg)	610	610	610	610	610	610	610	610	610	610

AZT is Zidovudine, Formulation F1 was matrix tablets prepared using 15% HPMC K4 M; formulation F2 was matrix tablets prepared using 20% HPMC K4 M; formulation F3 was matrix tablets prepared using 25% HPMC K4 M; formulation F4 was matrix tablets prepared using 15% Carbopol 934; formulation F5 was matrix tablets prepared using 20% Carbopol 934; formulation F6 was matrix tablets prepared using 25% Carbopol 934; formulation F7 was matrix tablets prepared using HPMC K4 M and ethyl cellulose (1:1) 25%; formulation F8 was matrix tablets prepared using Carbopol 934 and ethyl cellulose (1:1) 25%; formulation F9 was matrix tablets prepared using HPMC K4 M and ethyl cellulose (1:1.5) 25% and formulation F10 was matrix tablets prepared using Carbopol 934 and ethyl cellulose (1:1) 25%

### Estimation of drug content:

From each batch of prepared tablets, 10 tablets were collected randomly and powdered. A quantity of powder equivalent to 300 mg was transferred into a 100 ml volumetric flask, 100 ml of phosphate buffer pH 7.4 was added and the solution was sonication for about 30 min. The solution was made up to 100 ml with phosphate buffer pH 7.4, filtered and suitable dilutions were made with phosphate buffer pH 7.4. Same concentration of the standard solution was also prepared by taking 100 mg of drug in a 100 ml volumetric flask made up to volume with phosphate buffer pH 7.4. The drug content was estimated by measuring the absorbance of both standard and sample solutions at 265.6 nm using UV/Vis spectrophotometer (Systronics 2201).

### *In vitro* release studies:

The *in vitro* dissolution studies were performed using USP XXIV dissolution apparatus No.2 (paddle) type at 50 rpm. The dissolution medium consisted of 0.1 N hydrochloric acid for first 2 h and for subsequent 10 h in phosphate buffer pH 7.4 (900 ml), maintained at 37±0.5°. The release studies were conducted in triplicate. Aliquots of samples (5 ml) were withdrawn at specific time intervals and drug content was determined spectrophotometrically at 265.6 nm. The *in vitro* release data obtained was treated to zero order rate equation, first order rate equation, Higuchi’s equation^8^ (Q = Kt^1/2^), to know precisely the mechanism of drug release from the matrix tablet.

## RESULTS AND DISCUSSION

**Fig. 1:**
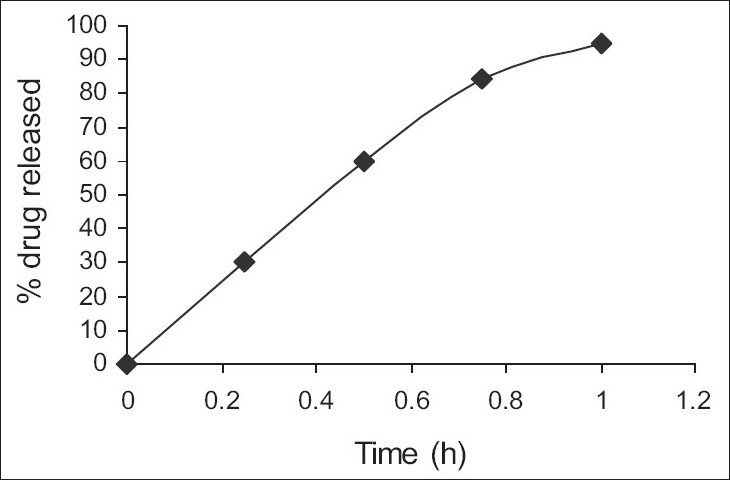
Dissolution profile of marketed tablet Marketed tablet (–♦–) used was Zidovir, Cipla Ltd, Mumbai, India

**Fig. 2:**
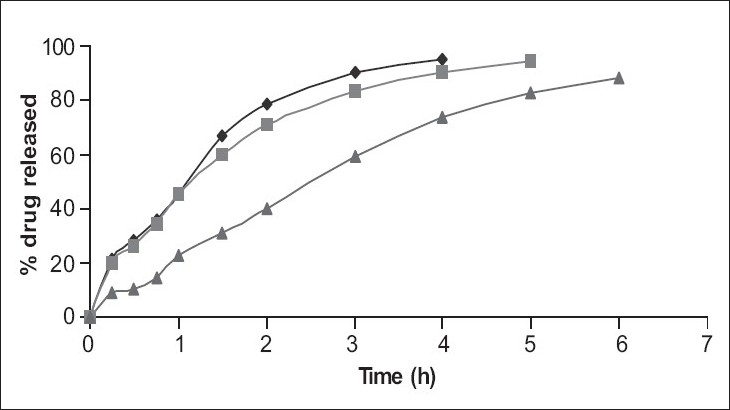
Dissolution profile of formulations F1, F2 and F3 F1 were matrix tablets prepared using 15% HPMC K4 M (–♦–); F2 were matrix tablets prepared using 20% HPMC K4 M (–■–) and F3 were matrix tablets prepared using 25% HPMC K4 M (–▲–)

**Fig. 3:**
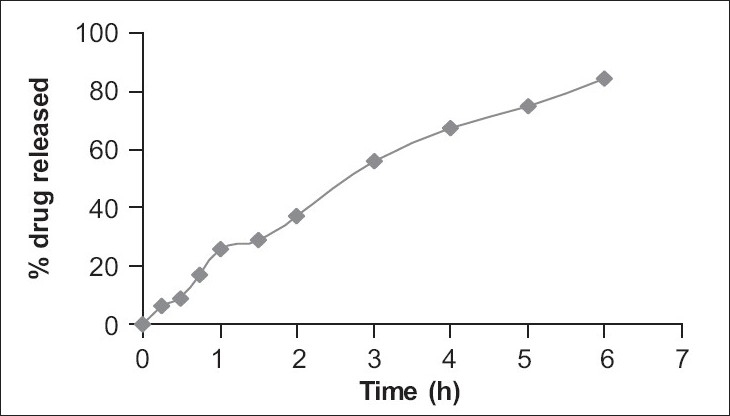
Dissolution profile of formulations F6 F6 were matrix tablets prepared using 25% Carbopol 934 (–♦–)

Fig. 1 shows the effect of different concentrations of HPMC K4 M 15%(F1), 20% (F2) and 25% wt/wt of tablet (F3) on% release (95.43%, 94.68% and 88.36% within 4 h, 5 h and 6 h of dissolution study, respectively) of AZT. The drug release was slower from the tablets containing HPMC K4 M as compared with that from marketed tablets (fig. 2). No significant difference in release rate was observed between tablets containing either 15% or 20% of HPMC K4 M. Drug release was decreased significantly when 25% of HPMC K4 M was used in formulation F3. However, 40 – 50% of drug was released in first 2.5 h of dissolution. In the *in vitro* study of matrix tablets containing 15%, 20% and 25% of Carbopol 934 (F4, F5 and F6), it was observed that tablets containing 15% and 20% were eroded within 20 min of the dissolution study indicating insufficient quantity of Carbopol 934 to form the gelatinous layer around the tablet core. Tablets containing 25% of Carbopol 934 were able to form the gelatinous layer around the tablet core and the drug release was found to be 86.45% within 6 h of dissolution study. However 40% - 60% of drug was released in the first 2.5 h of dissolution study (fig. 3).

Therefore, in the next batch of tablets, to control the initial burst release ethyl cellulose was included in the matrix in the ratio of 1:1 along with HPMC K4 M (F7) or Carbopol 934 (F8) which resulted in extending the drug release for a period of 12 h indicating fair uniform drug release through out the dissolution period (fig. 3). This may be due to a more rigid complex formed by hydrophilic polymers (HPMC K4 M and Carbopol 934) in presence of ethyl cellulose, which helped in retaining the drug in the matrix and did not allow rapid diffusion of soluble drug from the matrix. AZT has a very narrow (0.4–4.0 μM) therapeutic index. The commercially available marketed tablet produces initial high plasma concentration because of the absence of the release retardants, which may cause unwanted toxic effects like bone marrow depression that sometimes leads to withdrawal of drug therapy. Hence, it is essential to maintain the plasma level within the therapeutic index to eliminate toxic effects. The tablets formulated using combination of HPMC K4 M or Carbopol 934 and ethyl cellulose did not show any burst release indicating reduced possibility of dose dependent toxicity. The hydrophobic nature of ethyl cellulose seems to have contributed toward reduction in the penetration of the solvent molecules into the matrix.

Increasing the concentration of ethyl cellulose shows more retardation in the release of the drug from the formulation (F9) and (F10) containing ethyl cellulose in combination with HPMC K4 M and Carbopol 934 in the ratio 1:1.5, respectively. F9 showed 36% and F 10 showed only 30% of drug release in 12 h of dissolution study which may result in insufficient therapeutic concentration. Considering the decrease in the therapeutic effect on lowering the concentration of hydrophilic polymers over the dissolution release, the effect of only ethyl cellulose was not carried out.

**Fig. 4:**
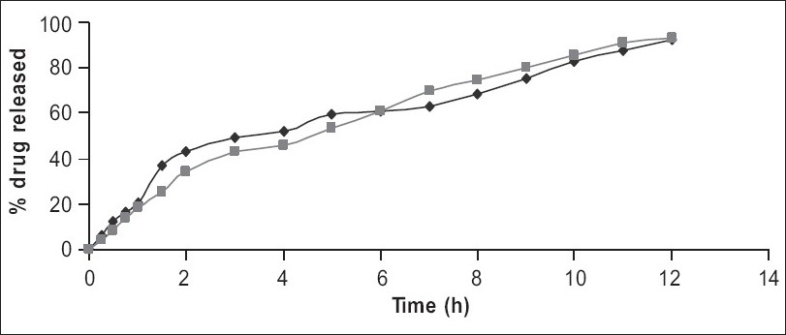
Dissolution profile of formulations F7, and F8 F7 were matrix tablets prepared using HPMC K4 M and ethyl cellulose (1:1) 25% (–♦–) and F8 were matrix tablets prepared using Carbopol 934 and ethyl cellulose (1:1) 25%

To know the kinetics of drug release, the data was treated according to different models. Drug release data of marketed tablet was fitted in zero order equation (r^2^=0.988) while that of F1, F2, F3, F6 formulations were fitted to first order equation (0.9954, 0.999. 0.989, 0.9841, respectively). Tablets of F7 and F8 formulation showed best fit into Higuchi equation (0.986, 0.9902, respectively) and indicated diffusion mechanism of drug release (fig. 4).

The swelling of tablets with HPMC K4 M or Carbopol 934 alone or in combination with ethyl cellulose can be attributed to the inherent property of hydrophilic polymers when in contact with aqueous dissolution fluids.

**Fig. 5:**
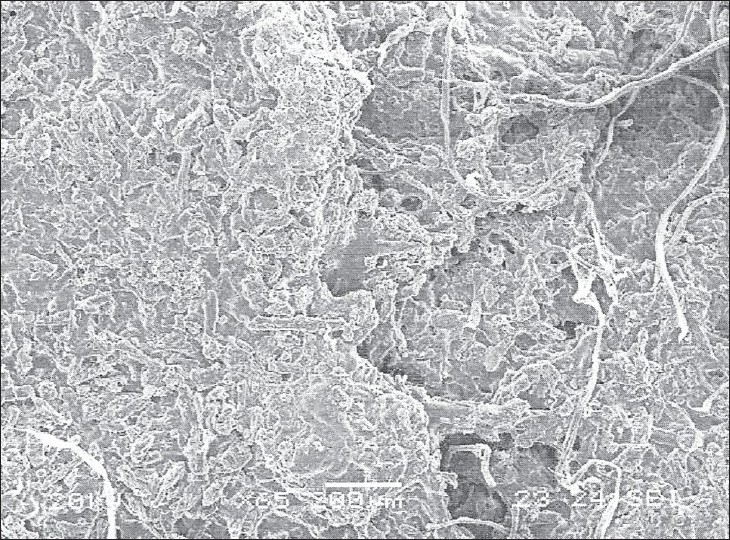
SEM photo of swelled tablet after 2 h dissolution

**Fig. 6:**
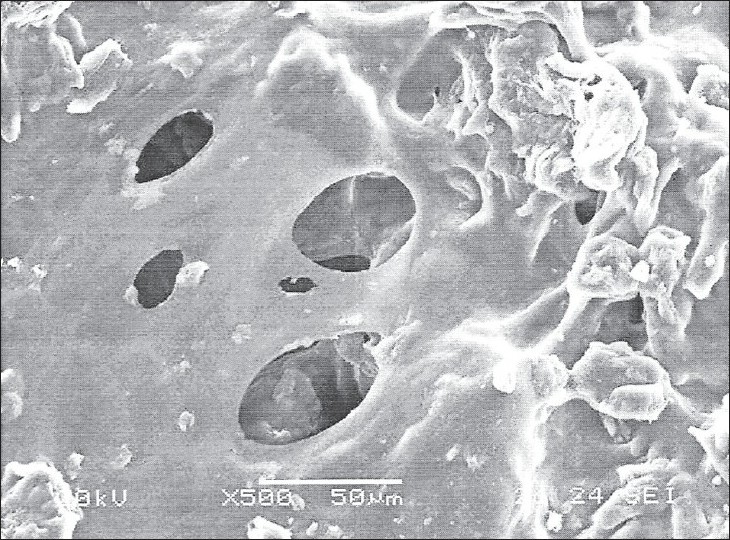
SEM photo of swelled tablet after 4 h dissolution

**Fig. 7:**
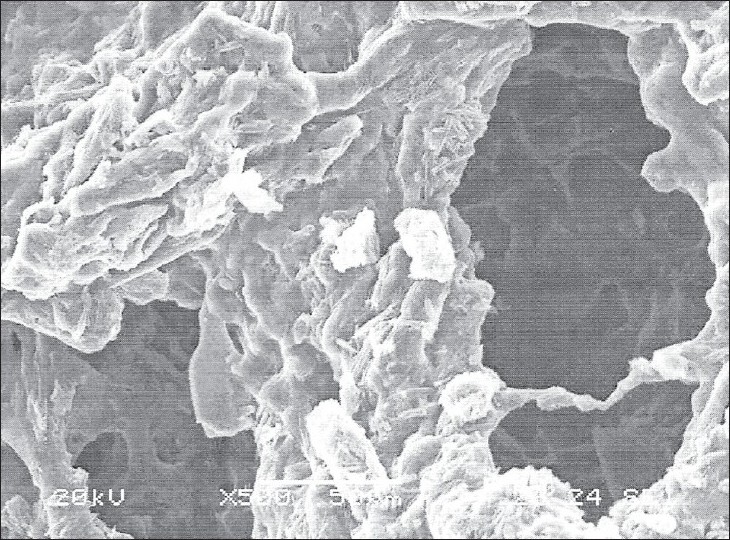
SEM photo of swelled tablet after 8 h dissolution

SEM study further confirmed both diffusion and erosion mechanisms to be operative during drug release from the optimized batch of matrix tablet (F7 and F8). SEM photomicrograph of the matrix tablet taken at different time intervals after the dissolution experiment showed that matrix was intact and pores had formed throughout the matrix (fig. 5). SEM photomicrographs of tablet surface at different time intervals also showed that erosion of matrix increased with respect to time indicated by the photomicrographs at 2, 4 and 8 h revealing pores with increasing diameter. These photomicrograph also revealed the formation of gelling structure indicating the possibility of swelling of matrix tablets (figs. 6 and 7). Hence, the formation of both pores and gelling structure on tablet surface indicates the involvement of both erosion and diffusion mechanisms to be responsible for sustaining the release of AZT from formulated matrix tablets.

Overall it can be concluded that, the presence of ethyl cellulose as well as the% of total matrix material significantly influenced the release rate of drug. The tablets with 25% of matrix material (1:1 ratio of HPMC K4 M or Carbopol 934 and ethyl cellulose) gave satisfactory results on the formulation of controlled release dosage form of AZT. Further, the controlled release of AZT at specific ratios of hydrophilic and hydrophobic polymers may be confirmed by continuing the studies for *in vivo* release.
